# Photoacoustic image improvement based on a combination of sparse coding and filtering

**DOI:** 10.1117/1.JBO.25.10.106001

**Published:** 2020-10-07

**Authors:** Ebrahim Najafzadeh, Parastoo Farnia, Saeedeh N. Lavasani, Maryam Basij, Yan Yan, Hossein Ghadiri, Alireza Ahmadian, Mohammad Mehrmohammadi

**Affiliations:** a Tehran University of Medical Sciences, Medical Physics and Biomedical Engineering Department, Faculty of Medicine, Tehran, Iran; b Tehran University of Medical Sciences, Research Centre of Biomedical Technology and Robotics, Imam Khomeini Hospital Complex, Tehran, Iran; c Shahid Beheshti University of Medical Sciences, Department of Biomedical Engineering and Medical Physics, Faculty of Medicine, Tehran, Iran; d Wayne State University, Department of Biomedical Engineering, Detroit, Michigan, United States; e Tehran University of Medical Sciences, Research Center for Molecular and Cellular Imaging, Tehran, Iran; f Wayne State University, Department of Electrical and Computer Engineering, Detroit, Michigan, United States

**Keywords:** photoacoustic imaging, signal denoising, sparse coding, total variation, filtering, signal-to-noise ratio

## Abstract

**Significance:** Photoacoustic imaging (PAI) has been greatly developed in a broad range of diagnostic applications. The efficiency of light to sound conversion in PAI is limited by the ubiquitous noise arising from the tissue background, leading to a low signal-to-noise ratio (SNR), and thus a poor quality of images. Frame averaging has been widely used to reduce the noise; however, it compromises the temporal resolution of PAI.

**Aim:** We propose an approach for photoacoustic (PA) signal denoising based on a combination of low-pass filtering and sparse coding (LPFSC).

**Approach:** LPFSC method is based on the fact that PA signal can be modeled as the sum of low frequency and sparse components, which allows for the reduction of noise levels using a hybrid alternating direction method of multipliers in an optimization process.

**Results:** LPFSC method was evaluated using in-silico and experimental phantoms. The results show a 26% improvement in the peak SNR of PA signal compared to the averaging method for in-silico data. On average, LPFSC method offers a 63% improvement in the image contrast-to-noise ratio and a 33% improvement in the structural similarity index compared to the averaging method for objects located at three different depths, ranging from 10 to 20 mm, in a porcine tissue phantom.

**Conclusions:** The proposed method is an effective tool for PA signal denoising, whereas it ultimately improves the quality of reconstructed images, especially at higher depths, without limiting the image acquisition speed.

## 1 Introduction

In the last two decades, photoacoustic imaging (PAI) as a non-invasive hybrid imaging modality has been used in a wide range of preclinical and clinical applications, such as functional brain mapping,^1^^,^^2^ molecular imaging,^3^^,^^4^ cancer diagnosis and staging,^5^^–^^8^ tissue vasculature imaging,^9^^–^^12^ guiding interventional procedures,^13^^,^^14^ and dental health.^15^^,^^16^ PAI detects the optical absorption contrast in tissue through the conversion of light to heat and thermoelastic effect, leading to the generation of acoustic waves.^17^^–^^19^ When the tissue is illuminated by short light pulses, the endogenous chromophores, such as hemoglobin, generate a photoacoustic (PA) signal due to their optical absorption.^20^^,^^21^ In this procedure, the light energy is transformed into acoustic waves, and the efficacy of this conversion is often affected by the presence of noise arising from the surrounding background.^22^^,^^23^ Therefore, the PA signal is often mixed by background noise, including thermal-acoustic noise in the medium as well as the transducer and electronic noises.^24^ White Gaussian noise is one of the most common models for these types of randomly distributed thermal and electronic noise.^25^ However, there is another component of noise arising from the light attenuation phenomena, caused by scattering characteristics of the tissue.^26^ Furthermore, other types of noise disturb the PA signals, such as the fixed-pattern noise caused by electromagnetic interference. The combination of these different types of noise in the PA signal leads to a low signal-to-noise ratio (SNR) and results in a poor quality reconstructed PA image.^27^^–^^30^

Several previously reported studies have attempted to improve the image reconstruction algorithms to achieve noise- and artifact-free PA images. However, acquiring high-quality PA images from noisy signals requires an effective denoising technique,^31^^–^^36^ prior to utilizing reconstruction algorithms. The most commonly used technique to reduce the noise level and improve the SNR of the PA signal is frame averaging, where the SNR improvement is proportional to the square root of the number of averaged frames.^37^ However, the signal averaging method needs to acquire multiple frames, which is time-consuming and affects the frame rate of PA imaging.^29^ Alternatively, the adaptive filtering method without any prior knowledge requirement was proposed for low-energy pulse laser diodes PA signal enhancement.^28^ They average fewer frames, in comparison with conventional averaging techniques, which leads to shorter acquisition time.^28^ Notwithstanding, the requirement of frames averaging in the latter method compromises the imaging speed.

Linear time-variant filtering techniques have been widely used to improve the SNR of the PA signal. Generally, these filtering techniques suffer from the inability of low-pass and bandpass filters when signal and noise share similar frequency spectrum.^38^ A commonly used PA signal denoising technique is the wavelet denoising method.^39^^–^^43^ Wavelet-based denoising methods face some challenges, such as choosing an appropriate basis function, the optimum number of wavelet decomposition levels, and especially choosing an optimum threshold value.^28^ While there are solutions for these drawbacks, these solutions are often complicated and computationally expensive.^44^^,^^45^ Furthermore, different denoising methods based on empirical mode decomposition (EMD)^46^ were proposed to improve the SNR of the signal. The EMDs decompose the PA signal into several intrinsic mode functions, which should be selected expertly and used for signal denoising. In this regard, the combination of the EMD method and mutual information (MI) was proposed to denoise PA signals.^29^ Although this method outperforms conventional wavelet and bandpass filtering methods in the term of SNR, it works based on an inaccurate assumption that high-frequency intrinsic mode functions contain considerable noise, and low-frequency intrinsic mode functions contain the majority of useful signals. Additionally, it is not an appropriate technique in the process of real-time PAI imaging, considering EMD and MI consumed time.

Since the size of the light absorber defines the spectrum content of the PA signals, these signals are usually broadband and cannot be considered as signals with specific frequency bands.^47^ On the other hand, the PA signal could be considered as a sparse signal with a sparse derivative. Therefore, one can model the PA signal as a sum of two components of low frequency and sparse.

In this study, for the first time, we proposed an approach for PA signals denoising based on a combination of low-pass filtering and total variation (TV) denoising, allowing for using a hybrid alternating direction method of multipliers (ADMM) in the optimization processes. Since the proposed method can be defined as a combination of low-pass filtering and sparse coding, we call it LPFSC.

The rest of this paper is organized as follows. Section 2 discusses the theory of TV denoising and ADMM methods, which are used in this paper. Section 3 describes the proposed denoising method, validation studies, and evaluation criteria. The experiments conducted to evaluate the performance of the proposed approach are described in Sec. 3, and the results are discussed in Sec. 4, followed by conclusions in Sec. 5.

## 2 Theoretical Background

### 2.1 Principles of Total Variation Denoising

Recently, the TV approach that promotes the sparsity of signals in the gradient domain has attracted significant attention in signal denoising applications.^48^ The goal of TV denoising technique is to efficiently estimate and recover the desired N-point signal $S={\left\{s(n)\right\}}_{n=1}^{N}$ with the sparse or sparse-derivative representation from the measured noisy signal x, which is defined as

x(n)=s(n)+w(n), (1)

where w(n) is considered as additive Gaussian noise with the variance of $\sigma^{2}$. TV denoising could be defined as the constrained minimization problem of a non-differentiable cost function in terms of the $l_{1}$ norm as below:

$$\arg\;\underset{s}{\min}\;{\left\|Ds\right\|}_{1},\;\text{subject to},\;{\left\|x-s\right\|}_{2}^{2}\leq N\sigma^{2},$$ (2)

where D as the first-order difference matrix is of size N×(N−1) and Ds is the first-order difference of an N-point signal s(n).^49^ With proper regularization parameter selection, Eq. (2) could be converted to the unconstraint problem as

$$Tvd(x,\lambda )=\arg\;\underset{s}{\min}\{\frac{1}{2}{\left\|x-s\right\|}_{2}^{2}+\lambda {\left\|Ds\right\|}_{1}\}.$$ (3)

In this optimization problem, the regularization parameter λ plays a significant role and controls the degree of smoothing, so that when the λ=0, there is no smoothing and the result is the same as minimizing the sum of squares. On the other hand, increasing λ assigns a higher weight to the second term of Tvd(x,λ), which measures the oscillation of the desired signal s(n) and makes the solution s(n) piecewise. Although there are different algorithms to solve the TV denoising problem, majorization–minimization (MM) is found suitable to solve the optimization problem, which is hard to solve directly.^50^

### 2.2 Alternating Direction Method of Multipliers

ADMM is a simple but powerful algorithm to solve a convex optimization problem by breaking it into smaller subproblems.^51^^,^^52^ The ADMM algorithm is designed to solve the separable convex problems of the form:

min f(x)+g(y),subject to  Ax+By=c, (4)

where $x\in R^{n}$, $y\in R^{m}$, $A\in R^{p\times n}$, and $B\in R^{p\times m}$. The augmentation Lagrangian in Eq. (4) can be written as

$$L_{\rho }(x,y,\lambda )=f(x)+g(y)+\lambda^{T}(Ax+By-c)+(\frac{\rho }{2}){\left\|Ax+By-c\right\|}_{2}^{2},$$ (5)

where ρ is the penalty parameter, which is considered positive, and λ is the Lagrangian multiplier. Equation (5) is solved using three steps: x-minimization and y-minimization, which are split into N separate problems, and an updating step for multiplier λ as follows:

$$x^{k+1}≔\arg\;\underset{x}{\min}\;L_{\rho }(x,y^{k},\lambda^{k}),\;y^{k+1}≔\arg\;\underset{y}{\min}\;L_{\rho }(x^{k+1},y,\lambda^{k}),\;\lambda^{k+1}≔\lambda^{k}+\rho (Ax^{k+1}+By^{k+1}-c).$$ (6)

## 3 Method and Materials

### 3.1 Low-Pass Filtering and Sparse Coding

To solve the problem of PA signal denoising, the PA signal is modeled as the measured noisy signal x(n), which is defined as

$$x(n)=s_{\text{lowfrq}}(n)+s_{\text{sparse}}(n)+w(n),$$ (7)

where the desired signal s(n) includes two main components, a low-frequency component and a sparse or a sparse-derivative component. Here, $s_{\text{lowfrq}}(n)$ represents the low-frequency component of the desired signal, $s_{\text{sparse}}(n)$ represents the sparse or the sparse-derivative components of the desired signal, and w(n) is considered as additive Gaussian noise with the variance of $\sigma^{2}$. Since we are looking for efficient estimation of $s_{\text{sparse}}(n)$ and $s_{\text{lowfrq}}(n)$, considering Eq. (7), in the following of $s_{\text{sparse}}(n)$ estimation, one can estimate $s_{\text{lowfrq}}(n)$ as follows:

slowfrq(n)≈lowpass[x(n)−s^sparse(n)], (8)

By replacing $s_{\text{lowfrq}}(n)$ based on Eq. (7), Eq. (8) could be written as

x(n)−w(n)−ssparse(n)≈lowpass[x(n)−s^sparse(n)], (9)

where $s_{\text{sparse}}(n)$ and s^sparse(n) are approximately equal. Therefore, Eq. (8) could be modified to

[x(n)−s^sparse(n)]−lowpass[x(n)−s^sparse(n)]≈w(n), (10)

Considering the assumption that the frequency response of the low-pass filter is approximately zero-phase, we can conclude that

highpass[x(n)−s^sparse(n)]≈w(n), (11)

where high pass refers to the high-pass filter. To achieve a computationally efficient approach, a zero-phase non-causal recursive high-pass filter which is proposed in Ref. 49 was deployed in our method. Since the PA signal is sparse and has a sparse derivative, the cost function of the optimization problem contains a linear combination of two regularization parameters, which promote piecewise smooth and sparse solutions. Therefore, the denoising problem, shown in Eq. (3), can be expressed as the unconstraint minimization problem of a non-differentiable cost function in terms of $l_{1}$ norm as below:

$$\arg\;\underset{s,s_{\text{sparse}}}{\min}\{\frac{1}{2}{\left\|\text{highpass}(x-s_{\text{sparse}})\right\|}_{2}^{2}+\lambda_{0}{\left\|s\right\|}_{1}+\lambda_{1}{\left\|Ds\right\|}_{1}\}.$$ (12)

There are many solutions, such as MM and ADMM, to solve Eq. (12). Since the denoising process and image reconstruction speed are vital to achieve real-time PA imaging, we proposed to use the hybrid consensus ADMM method^53^ to achieve a linear convergence and accelerating the conventional ADMM.

### 3.2 Validation Studies

#### 3.2.1 In-silico study

A simulation study generated by the k-wave toolbox in MATLAB^®^ (Mathworks, Massachusetts) was performed to evaluate the performance of LPFSC in PA signal denoising.^54^ The initial pressure distribution is given by a 512×512  pixel image representative of a vascular structure. A 10-mm square grid was created, and the circular array detectors with 9-mm diameter and 60 elements, evenly spaced, were located around the region of interest (ROI) to receive the propagated PA wave from the object. We considered the center of each sensor as a point source. The sound speed was considered to be 1500  m/s. The corresponding time array has 1019 data points that are 9.259 ns apart from each other (108-MHz sampling frequency). The input size of the PA signal was assigned 1019×60, and the reconstructed images have 128×128  pixels.

#### 3.2.2 Experimental PA data acquisition setup

**Fig. 1:**
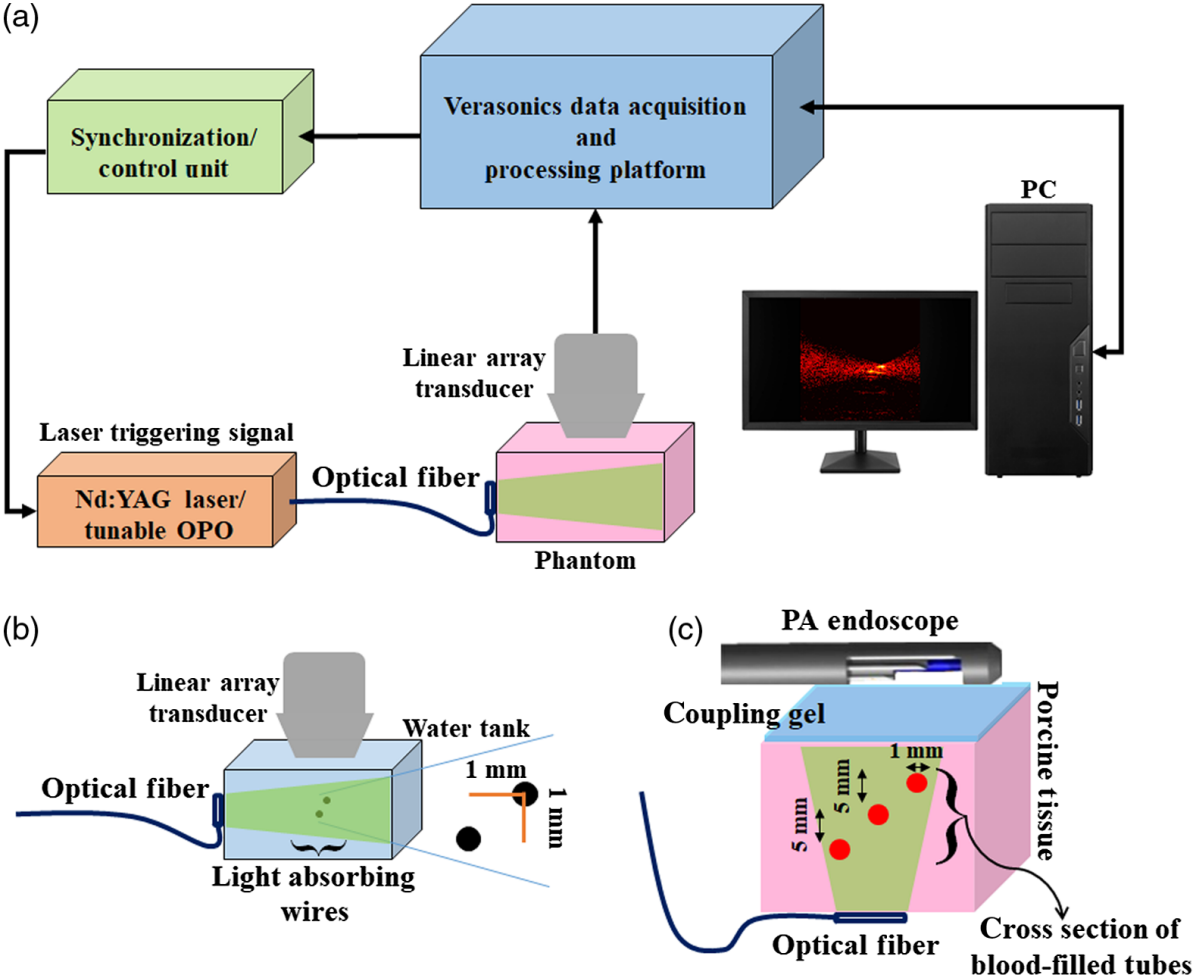
Schematic of (a) the experimental setup used for the PA imaging of (b) two light-absorbing filaments that were placed inside a water tank and (c) cross-section view of blood-filled tubes embedded in a porcine tissue phantom.

**Fig. 2:**
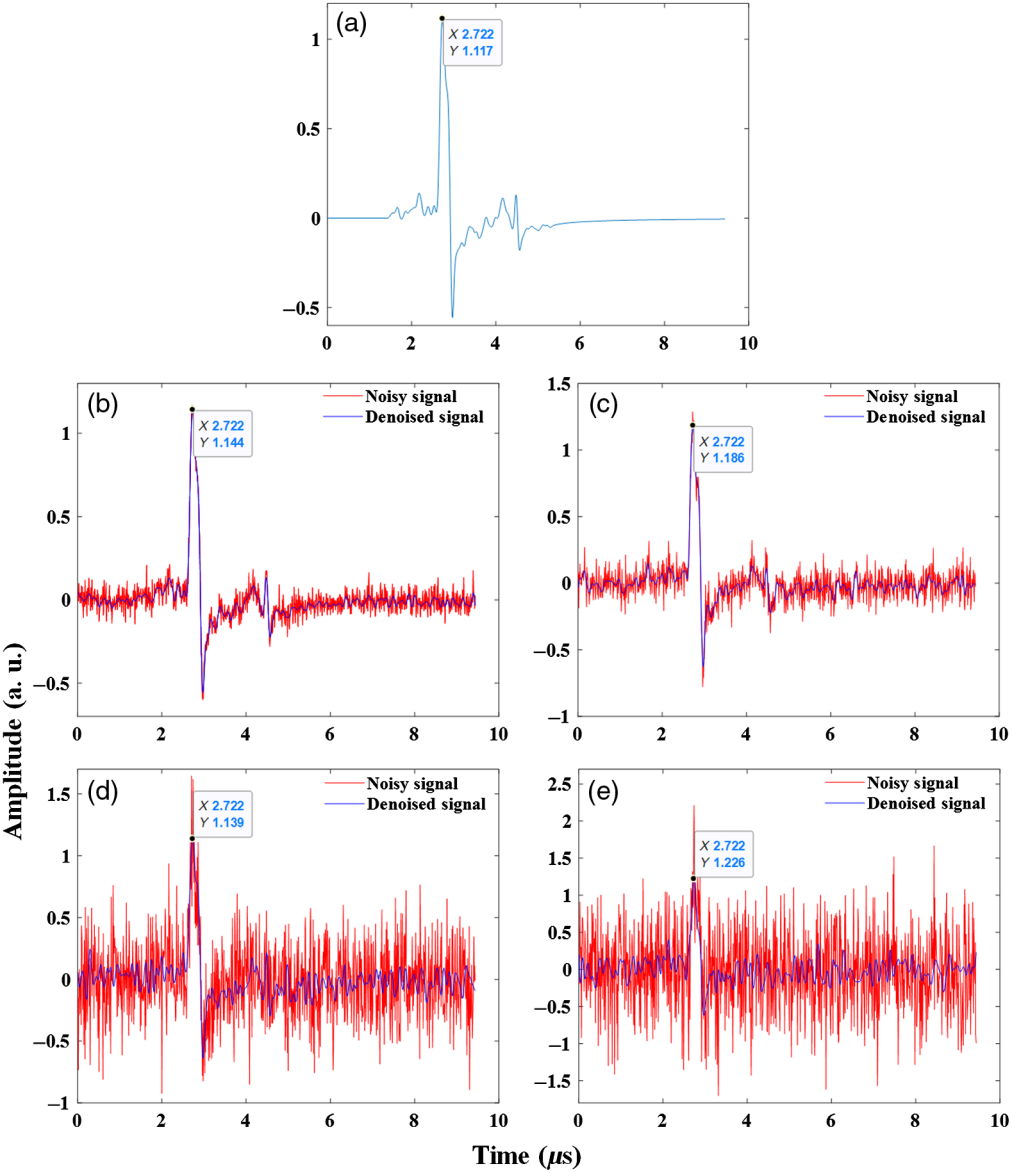
The denoising results of simulated PA signal in four different levels of noise. Noisy signals are shown in red and denoised signals are depicted in blue. (a) The original simulated PA signal detected on a detector #50, (b) SNR: 10 dB (c) SNR: 5 dB, (d) SNR: −5  dB, and (e) SNR: −10  dB. The horizontal and vertical axes indicate the time coordinate and amplitude of the recovered signal in different noise levels, respectively.

To further evaluation of the proposed PA signal denoising method and its effects on the quality of reconstructed PA images, phantom experiments were performed. The designed phantoms and the imaging setups are shown in Fig. 1. The first phantom contains two light-absorbing filaments with a diameter of 150  μm that were placed 1 mm apart from each other inside a water tank [Fig. 2(b)]. An Nd: YAG/OPO nanosecond pulsed laser (Phocus core system, OPOTEK Inc., Carlsbad, California) with the pulse repetition rate of 10 Hz at wavelengths of 680 nm was used to illuminate the phantom. An ultrasound scanner (US) (Vantage 128TM, Verasonics Inc., Kirkland, Washington) with a 128 elements linear array transducer (L114v, Verasonics, Inc., Kirkland, Washington) was used to receive the propagated PA RF data [Fig. 1(a)].

The second phantom contains three polytetrafluoroethylene tubes (1-mm diameter), filled with human blood and embedded within a 30-mm-thick porcine tissue background. Blood-filled tubes were placed inside the porcine tissue background at different depths from about 10 to 20 mm with 5-mm increments. PA acquisition was performed with the laser energy of 3  mJ/pulse at the wavelength of 680 nm. A fiber bundle with a diameter of 20 mm used for guiding the laser light to the tissue. A 64-element phased-array US endoscopic transducer, with an active aperture of 9-mm long, was coupled to the phantom to acquire acoustic signals and provide high-resolution sector images [Fig. 1(c)].

### 3.3 PA Signal Evaluation and Analysis

To evaluate the proposed approach, evaluation criteria such as peak signal-to-noise (PSNR) for signals, structural similarity index (SSIM), and contrast-to-noise ratio (CNR) for estimated images were used.

The PSNR as a common criterion to measure the quality of signal denoising based on the maximum possible value in the signal and mean square differences between denoised and reference signals is expressed in term of the logarithmic decibel scale (dB) as below:

$$\mathrm{PSNR}=20\;\log_{10}(\frac{S_{\max}}{\sqrt{\mathrm{MSE}}}),\;\mathrm{MSE}=\frac{1}{M}\overset{M-1}{\underset{m=0}{\sum }}{\left[S_{\text{original}}(m,1)-S_{\text{denoised}}(m,1)\right]}^{2},$$

where MSE is defined as a mean-square-error, $S_{\text{original}}$ and $S_{\text{denoised}}$ are original and denoised signals in size of M×1, respectively, and $S_{\max}$ is maximum possible value in signals.

We created PA images that represent an optical absorption distribution map of the targets via the conventional delay-and-sum (DAS) approach as the most commonly used reconstruction method in the PAI area.^55^ For reconstructed images, the SSIM (in a scale of 0 to 1) as one of the most common criteria for image quality assessment and for evaluating the similarity of images (i.e., reference image and reconstructed images) is defined^56^ as

$$\mathrm{SSIM}=\frac{\left(2\mu_{\text{Original}}\mu_{\text{estimated}}+c_{1})(2\sigma_{\text{original},\text{estimated}}+c_{2}\right)}{\sqrt{\left(\mu_{\text{original}}^{2}+\mu_{\text{estimated}}^{2}+c_{1})(\sigma_{\text{original}}^{2}+\sigma_{\text{estimated}}^{2}+c_{2}\right)}},$$

where $\mu_{\text{original}}$ and $\mu_{\text{estimated}}$ are the mean of the original and estimated images, respectively, and also $\sigma_{\text{original}}^{2}$ and $\sigma_{\text{estimated}}^{2}$ are the variances of the original and estimated images, respectively. It is worth to mention that $\sigma_{\text{original},\text{estimated}}$ is the covariance between the original and estimated image. The values of $c_{1}$ and $c_{2}$ are considered as constant values to avoid instability when the sum square of means or variances are very close to zero.

Finally, the CNR is a common criterion to determine image quality, especially in denoising processes with below definition:

$$\mathrm{CNR}=20\;\log(\frac{\left|S_{i}-S_{O}\right|}{\sigma_{O}}),$$

where $S_{i}$ and $S_{O}$ are the average intensity inside and outside of the objects, respectively. The $\sigma_{O}$ represents the standard deviation of the background. The background was defined as the pixels located inside the green dashed rectangular region selected in each set of PA images. For phantom studies, we considered the average of all frames as a ground truth (reference image) in each experiment.

## 4 Results and Discussion

To assess the proposed PA signal denoising method, we validated our method on numerical vessel phantom and experimental data of phantoms. The simulated PA signal, which was generated by the k-wave is shown in Fig. 2(a). Four different levels of additive Gaussian white noise with SNR levels 10, 5, −5, and −10  dB were added to the original simulated clean PA signal, and results of the proposed denoising method for the simulated noisy signals are shown in Figs. 2(b)–2(e). When the noise level was increased (for example, in SNR level −10), the PA signal peak was almost buried in added noise. As we have shown in Fig. 2, the LPFSC could suppress the noise and reconstruct the peak of the original signal, indicating the ability of the TV approach to recover the sparse or sparse-derivative signals. In the worst case (SNR level −10  dB), the difference between the original signal peak and the denoised signal peak is about 4%. This variance considering increasing the peak of noisy signal about 90% in comparison with the original signal is negligible. TV-based denoising is the most appropriate method for piecewise constant signals and preserves sharp edges in the underlying signal without requiring any step-size parameter as the amount of peak for the denoised signal.^57^ It is worth mentioning that the proposed method could recover denoised signal until the SNR decreased to −15  dB.

**Fig. 3:**
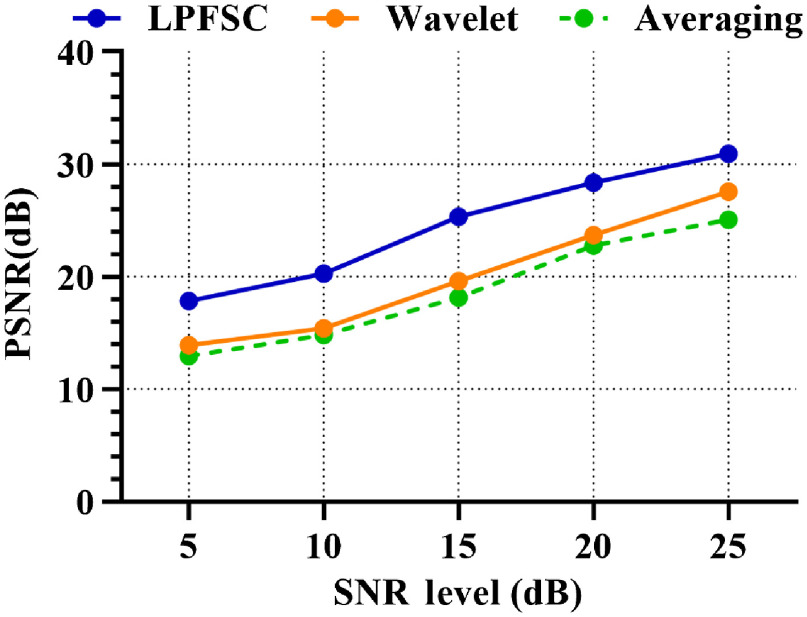
Comparison of three different denoising methods: wavelet-based signal denoising, the averaging method using 20 frames, and LPFSC in terms of PSNR improvements at five different noise levels. The results clearly demonstrate the superior performance of LPFSC compared to wavelet and averaging.

For further evaluation, the LPFSC signal denoising method was compared with two well-known and widely used approaches of averaging and wavelet-based denoising methods. As shown in Fig. 3, the PSNR of the LPFSC method was compared to the frame averaging (using 20 frames) and wavelet-based denoising methods, at different noise levels. For PA signal denoising by wavelet method, a commonly used Symlet 6 wavelet with six-level of decomposition and Stein’s Unbiased Risk Estimation threshold (SURE threshold) were selected.^29^

The quantitative results obtained with the simulations show that the LPFSC compensates the low SNR of PA signal and outperforms the competing wavelet and averaging denoising methods in terms of PSNR by 24% and 26%, respectively, across all simulated noise levels.

**Fig. 4:**
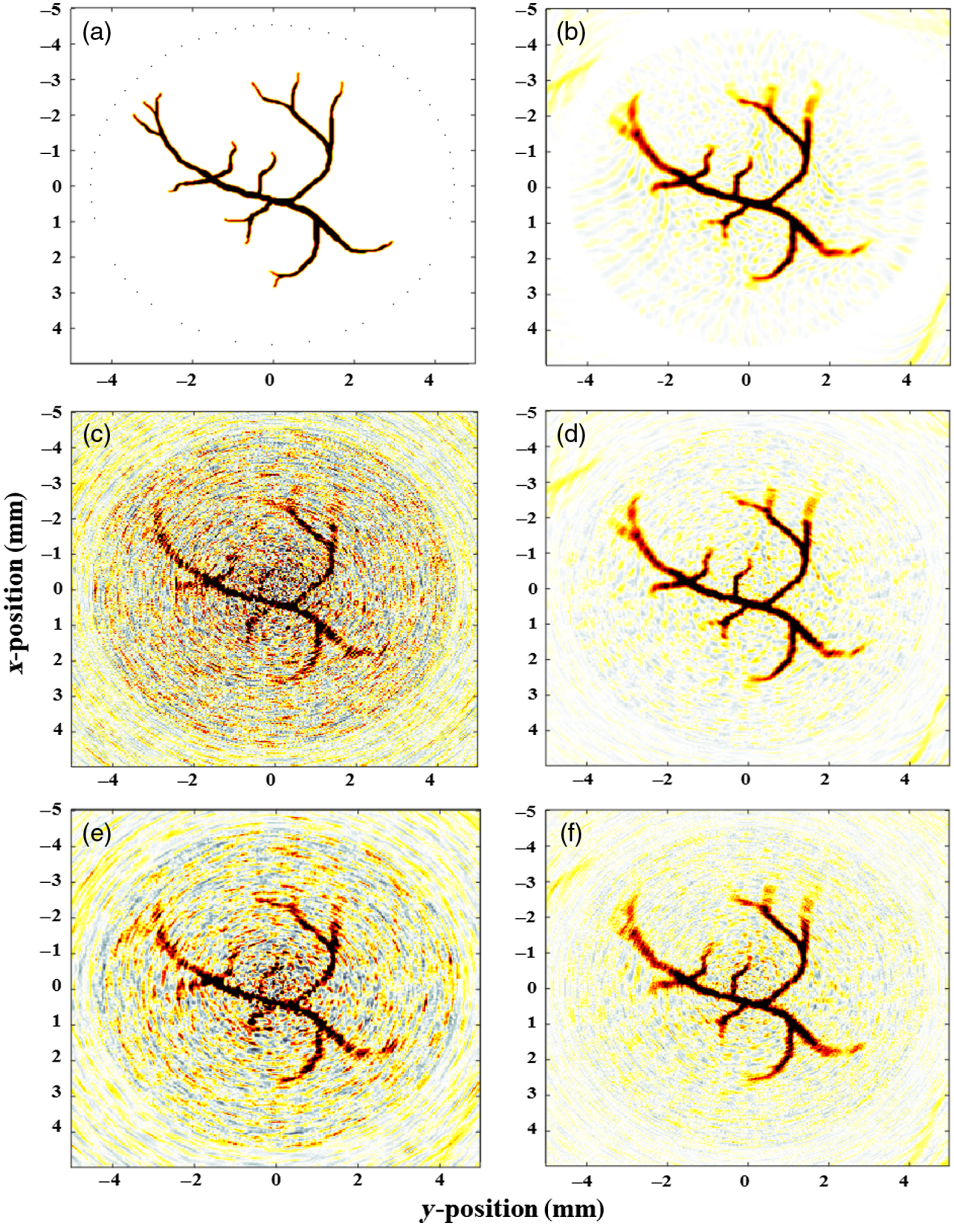
(a) Illustration of the phantom used for in-silico studies. Reconstructed images of: (b) simulated PA phantom, (c) noisy image when SNR is considered −10  dB, (d) PA image using the LPFSC denoising, (e) PA image using the wavelet denoised, and (f) PA image using the averaging of 20 frames for noise reduction.

The reconstructed images of denoised simulated PA signals with considering SNR of −10  dB for three methods of averaging, wavelet-based denoising, and LPFSC are shown in Fig. 4. The wavelet method has not been successful in recovering the small size vessels, which are corrupted by the noise. In addition, the averaging method using 20 frames cannot fully clean the signal due to presences of the coherent noise; however, our method using one frame could fully recover the original image and its details.

For the quantitative evaluation of the reconstructed images of denoised simulated PA signals, two SSIM and CNR criteria were used. Comparison between the results of three different methods, including wavelet-based signal denoising, averaging, and LPFSC in terms of SSIM and CNR with different levels of noise are shown in Table 1. On average (for different noise levels), the proposed denoising approach offers better CNR up to about 28% and 30%, and higher SSIM of about 23% and 24% in comparison with averaging and wavelet denoising methods, respectively. With increasing SNR levels from 5 to 20 dB and the reduction of noise level, the performance of all methods are improved. Although, for the lower SNR such as 5 dB, LPFSC outperforms wavelet and averaging denoising method by improvement about 58% and in terms of SSIM.

**Table 1:** Performance of three different signal denoising methods: wavelet-based, averaging method, and LPFSC across different levels of noise.

Level of SNR (dB)	Wavelet		Averaging		LPFSC	
	SSIM	CNR (dB)	SSIM	CNR (dB)	SSIM	CNR (dB)
5	0.59	65.01	0.58	67.23	0.93	87.61
10	0.78	72.53	0.79	72.98	0.95	94.53
15	0.86	75.15	0.87	76.23	0.98	96.70
20	0.86	76.66	0.91	77.12	0.99	98.21
Mean±std	0.77±0.13	72.33±5.17	0.78±0.14	73.39±4.47	0.96±0.03	94.26±4.68

**Fig. 5:**
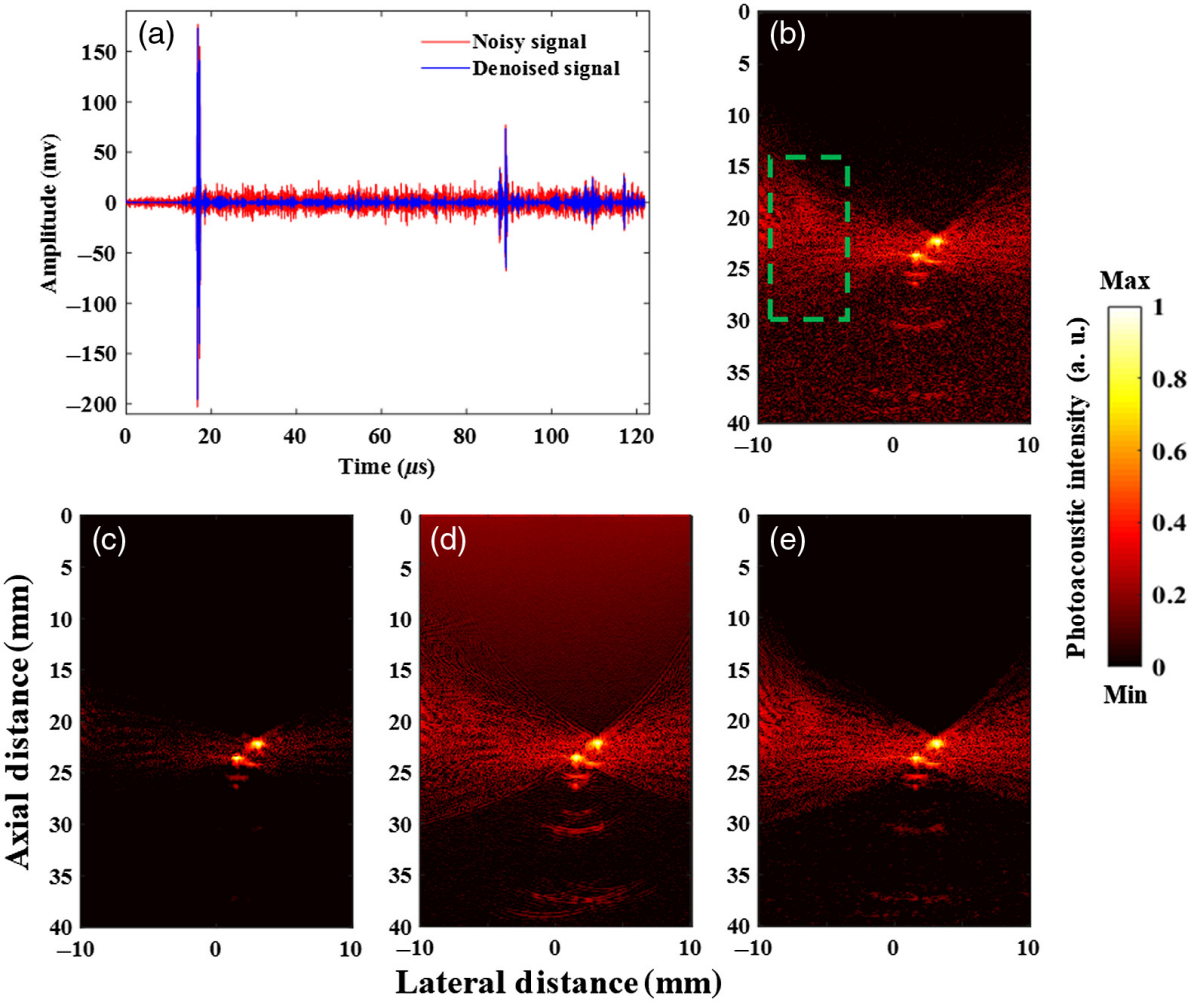
(a) The PA signal denoising of experimental phantom via LPFSC and (b) the original reconstructed image of one frame. The reconstructed images of phantom via three different signal denoising methods: (c) the LPFSC method (one frame), (d) the wavelet denoising method (one frame), and (e) the averaging all 20 frames. The background was defined as the pixels located inside the green dashed rectangular region.

During experimental studies, we evaluated our method in a set of phantom studies in which two small-sized absorbers were placed inside a water tank, illuminated from the side and images from the top using a US transducer operating at frequencies between 4 and 11 MHz. The PA signal denoising results for the acquired signals of different detectors from the 20th frames of phantom data are shown in Fig. 5. The experimental results of PA signal denoising show significant improvement for the PSNR of PA signal of wires phantom about 35% by recovering peaks of original signal and reduction of noise.

Additionally, reconstructed images of denoised PA signals through three different methods are shown in Fig. 5 and are compared in Table 2, in terms of SSIM, CNR, axial and lateral full width half maximum (FWHM) for experimental phantom data. The selection of FWHM is to demonstrate the geometrical accuracy of reconstructed PA images. The experimental results of PA signal denoising prove that the LPFSC beats the performance of averaging denoising method (using all 20 frames) as the reference in the term of SSIM and CNR criteria by the 17% and 38%. The LPFSC method provides a closer to real size reconstructed PA image with a mean of axial FWHM of 0.77±0.12  mm and lateral FWHM of 1.05±0.04  mm, for two objects in experimental data. In comparison with the reconstructed image of one frame without any signal denoising with axial FWHM of 0.85±0.18 and lateral FWHM of 1.08±0.05  mm, our method could preserve lateral FWHM and improve axial FWHM about 10%. Whereas the wavelet denoising method did not improve the lateral and axial FWHM, and the averaging method was not effective for axial FWHM. It is worth to mention that both of these methods improve CNR of the reconstructed image in comparison with the reconstructed image of one frame with CNR 19.32 dB. Therefore, one of the main improvements gained by the proposed LPFSC method is having reduced geometrical distortion as well as a high contrast at the same time. More importantly, the LPFSC method offers using one frame in comparison with the averaging method, which requires 20 frames. Using the hybrid ADMM, which stands out as efficient and easily implementable, leads to fast convergence of our method.

**Table 2:** Comparison of three methods of wavelet, averaging, and LPFSC method in terms of SSIM, CNR, and lateral and axial FWHM for the reconstructed image of the denoised signal.

Criteria	Wavelet	Averaging	LPFSC
SSIM	0.79	0.81	0.95
CNR (dB)	23.72	25.36	35.17
Lateral FWHM (mm) (mean + std)	1.20±0.06	1.11±0.09	1.05±0.04
Axial FWHM (mm) (mean + std)	1.12±0.16	0.98±0.06	0.77±0.12

**Fig. 6:**
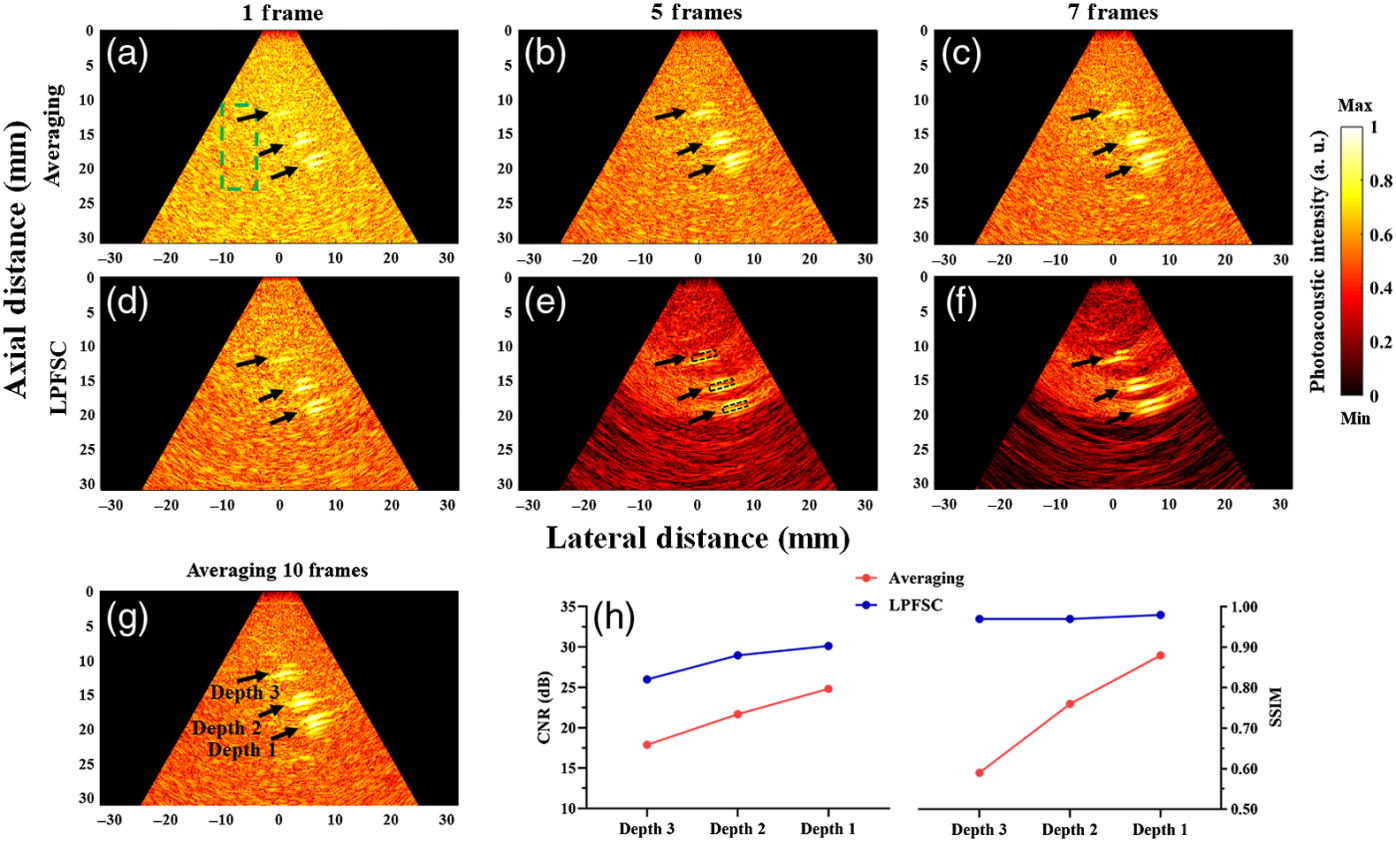
Reconstructed PA images of denoised signals of a porcine/blood inclusion phantom. (a)–(c) Frame averaging with 1, 5, and 7 averaged frames, respectively. (d)–(f) LPFSC with 1, 5, and 7 averaged frames, respectively. (g) Averaging 10 frames used as a reference. (h) The comparison of CNR and SSIM of two methods using five frames for inclusions located at different depths. The black arrows indicate objects located at depths 1, 2, and 3. Depths 1, 2, and 3 are about 20, 15, and 10 mm, respectively. The background was defined as the pixels located inside the green dashed rectangular region. The ROI for the target (object) is indicated with black dashed rectangular boxes in (e).

Finally, we evaluated the performance of the proposed denoising method with data acquired by an endoscopic probe and from the porcine tissue phantom. Since there was no significant difference between the results of frame averaging and wavelet method in in-silico study, the reconstructed PA images of denoised signal with frame averaging and LPFSC methods are compared in Fig. 6. The images form by averaging of 10 frames were used as a ground truth or the reference image. All constructed images from denoised signal using LPFSC and traditional frame averaging with the same number of frames (n=1 to 7) were compared to the ground-truth reference to calculate the SSIM. The number of averaged frames varied between one and seven. First and second rows related to the results of the averaging method and our proposed method, respectively. The walls of blood-filled tubes are generating strong PA signals that are visually presented as two parallel bright lines in PA images. To calculate CNR, an ROI located within the tube [as shown in Fig. 6(e)] was used to measure the signal from the target. To further quantify the image enhancements, CNR and SSIM of frame averaging and LPFSC using five frames are compared in Fig. 6(h). The use of five frames averaging in conjunction with LPFSC markedly reduced the background noise, which improved the mean of CNR and SSIM by 32% and 31%, respectively.

**Fig. 7:**
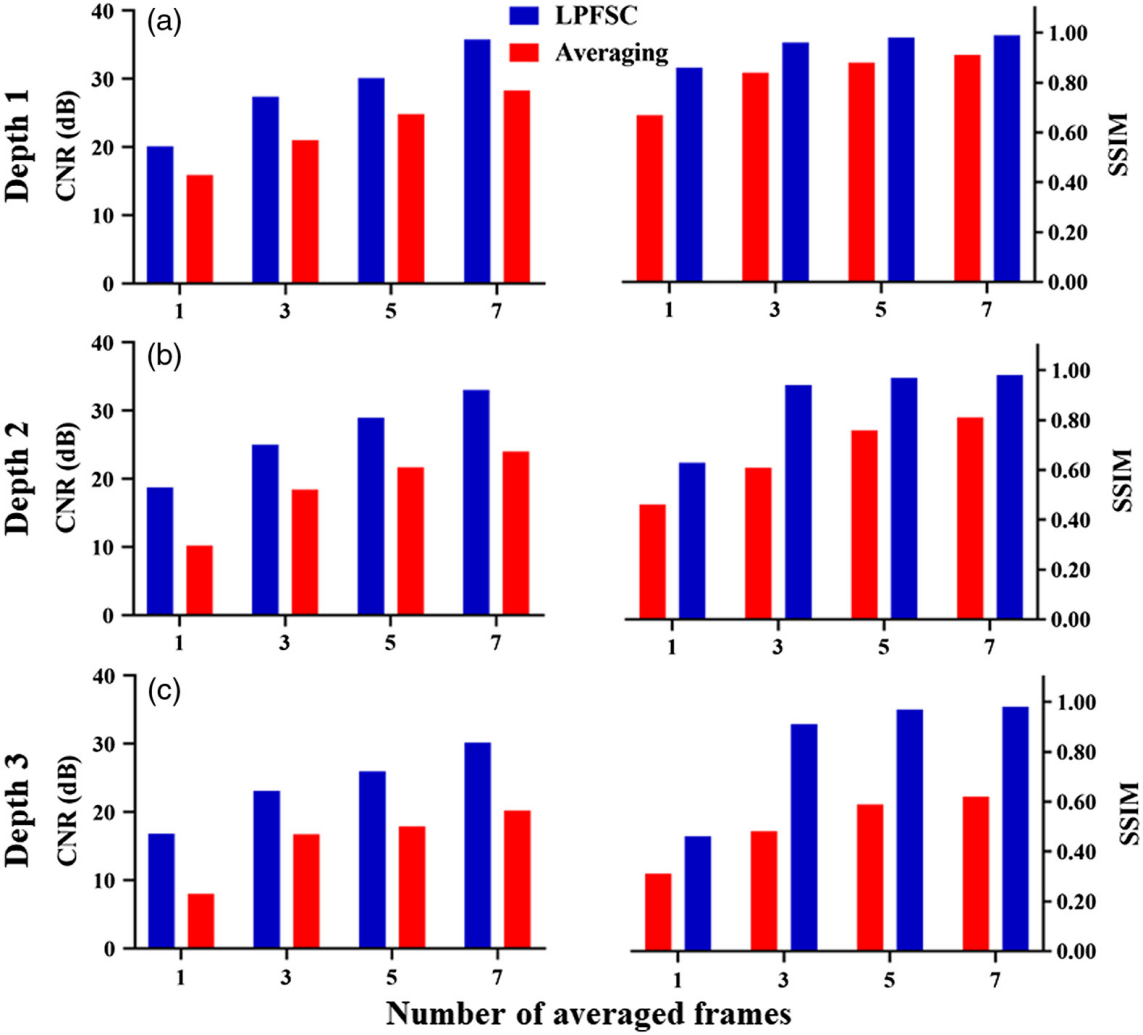
Porcine tissue results: comparison between the LPFSC with averaging using different number of averaged frames ranging from 1 to 7 in terms of CNR and SSIM: (a) depth 1, (b) depth 2, and (c) depth 3. CNR and SSIM parameters are evaluated and the results indicate the superior performance of LPFSC compared to averaging only.

Evaluation of CNR and SSIM for different numbers of averaged frames and at different imaging depths are shown in Fig. 7. As anticipated, CNR and SSIM parameters are improved with increasing the number of averaged frames. However, the proposed denoising method provides higher CNR and SSIM using the same number of averaged frames for objects placed at different depths. The mean of CNR using LPFSC with one frame was improved 63% in comparison with averaging for the objects evaluated at three different depths. A closer look at the results reveal that the mean CNR using the proposed denoising method using three averaged frames is higher than averaging only and using seven averaged frames. This comparison clearly shows the potential of using the proposed method to enhance the quality of PA images with a smaller number of averaged frames, which can lead to a preserving image quality at higher imaging speed. Our results indicated the LPFSC with three frames averaging is capable of detecting the objects located at depth 3 with SSIM of 0.91, which shows 90% improvement compared to the standard averaging method. The average measured SSIM for objects located at three different depths was calculated as 0.88±0.15 when LPFSC is utilized. This result indicates 33% improvement compared to averaging alone.

In this study, a range of cutoff frequencies was experimentally tested on data sets. The accepted range of cutoff frequencies was concluded to be between 0 and $0.3\;\frac{\text{cycles}}{\text{sample}}$ for different data sets, experimentally. For in-silico and first experimental phantom studies, the optimal value of cutoff frequency was selected $0.1\;\frac{\text{cycles}}{\text{sample}}$ and for porcine tissue data, it was selected $0.15\;\frac{\text{cycles}}{\text{sample}}$. Also, we consider equal weight for the TV and LPF; therefore, the lambda value was selected equal to be 1, experimentally. Changing lambda within the range of 0.8 to 1.2 showed acceptable results for PA signal denoising. For lambda smaller than 0.8, the amplitude of denoised signal is decreased compared to the original signal. The lambda bigger than 1.2 downgraded the performance of LPFSC. The performance of LPFSC could also be affected by the adjustment of low-pass filter cutoff frequency. However, small deviations from the ideal cutoff frequency up to 20% could be compensated by the sparse denoising part of the proposed algorithm.

## 5 Conclusion

The efficiency of PA imaging is routinely limited by the presence of background noise and suffering from low SNR, which resulted in the poor quality of the reconstructed images. Since the PA signals can be modeled as a sum of two low frequency and sparse components, we proposed a denoising approach that simultaneously estimates a low-pass and a sparse signal from an acquired noisy signal based on TV optimization approach, and using hybrid ADMM. Both in-silico and experimental work on tissue mimics were used to evaluate the performance of the proposed technique. The results demonstrated that the LPFSC method possess a superior performance to compensate the low SNR PA signals and offered a better CNR and SSIM for the reconstructed images compared to the frame averaging method. This comparison clearly shows the potential of using our proposed method to enhance the quality of PA images while maintaining high-speed imaging which is an essential need in many applications of PA imaging. In other words, the frame rate of PA images, which is always a challenge in real-time PA imaging can be significantly improved.
